# Establishment and validation of a survival prediction model for stage IV non-small cell lung cancer: a real-world study

**DOI:** 10.3389/fimmu.2025.1508721

**Published:** 2025-03-06

**Authors:** Keao Zheng, Junyan Zhang, Tingting Xu, Fangyu Li, Feng Li, Jing Zeng, Yimeng Guo, Zhiying Hao

**Affiliations:** ^1^ School of Pharmacy, Shanxi Medical University, Taiyuan, China; ^2^ Department of Affiliated Cancer Hospital, Shanxi Medical University, Taiyuan, China; ^3^ Department of Pharmacy, Shanxi Province Cancer Hospital/Shanxi Hospital Affiliated to Cancer Hospital, Chinese Academy of Medical Sciences/Cancer Hospital Affiliated to Shanxi Medical University, Taiyuan, China

**Keywords:** clinical predictive modeling, advanced non-small cell lung cancer, three-year survival, chemotherapy, immunotherapy

## Abstract

**Objective:**

The aim of this study is to develop and validate a predictive model for predicting survival in individual advanced non-small cell lung cancer patients by integrating basic patient information and clinical data.

**Methods:**

A total of 462 patients with advanced non-small cell lung cancer collected from Shanxi Cancer Hospital were randomly assigned (in a 7:3 ratio) to a training cohort and an internal validation cohort. Independent factors affecting patients’ 3-year survival were screened and predictive models were created by using a single-factor followed by multifactor Cox regression analysis. Evaluate the performance of the model using the consistency index (C-index), calibration curves, receiver operating characteristic curves (ROC) and decision curve analysis (DCA). The collected patients who received chemotherapy alone and those who received chemotherapy combined with immunotherapy were statistically paired using propensity score matching between the two groups, and subgroup analyses were performed among the screened variables.

**Results:**

A better prognostic model was created and a nomogram chart visualizing the model was drawn. Based on the median risk score of the training cohort, all individuals were categorized into high- and low-risk groups, with the high-risk group having worse OS in both cohorts (*P*<0.05). The results of subgroup analysis showed that chemotherapy alone versus chemotherapy combined with immunotherapy in patients with advanced NSCLC affected OS.

**Conclusion:**

A clinical predictive model was developed to predict 3-year survival in patients with advanced non-small cell lung cancer. The study demonstrated that chemotherapy combined with immunotherapy is superior to chemotherapy alone.

## Introduction

Lung cancer, as the leading cause of cancer-related deaths worldwide, poses a great threat to human health (1). Based on the size and type of cancer cells, lung cancer can be categorized into two types: small cell lung cancer (SCLC) and non-small cell lung cancer (NSCLC), of which NSCLC accounts for about 85% of lung cancers (2, 3). Lung cancer is subdivided into three types according to pathology: squamous cell carcinoma, lung adenocarcinoma, and large cell lung cancer (4). Due to the lack of obvious early symptoms, most NSCLC patients are in advanced stages upon diagnosis and have a poor prognosis (5). Faced with the high risk of surgical treatment for advanced NSCLC patients, radiotherapy and drug therapy are mostly used in clinical treatment (6). There are many drugs to choose from when receiving drug therapy programs, and the current main drug programs include traditional cytotoxic drug therapy, targeted drug therapy for tumor gene mutations, and emerging immune monoclonal antibody therapy (7, 8). Due to the long drug treatment cycle, it is also difficult to accurately assess the survival benefit of patients in clinical practice. In order to improve the therapeutic effect and the quality of patient survival, there is an urgent clinical need for a model that can predict the prognosis of patients with advanced NSCLC. Such a model can help physicians assess patients’ risk of disease progression, response to treatment, and survival expectations, and thus develop an individualized treatment plan for each patient. In this study, we constructed a survival model for advanced NSCLC patients treated with antitumor drugs can be used to assess the prognosis of advanced NSCLC patients and provide a reference for clinical treatment decisions.

## Method

### Patient selection

This retrospective study followed the guidelines of the Declaration of Helsinki and was approved by the Ethics Committee of Shanxi Cancer Hospital. The study was exempted from informed consent requirements. A total of 2005 cases of patients treated with antitumor drugs between December 2018 and May 2020 were queried for this study, and 462 patients diagnosed with advanced primary non-small cell lung cancer were finally included. Inclusion criteria: (1) Primary non-small cell lung cancer diagnosed at stage IV on initial admission. (2) Received anti-tumor drugs. (3) Clinical characterization and follow-up data can be used. Exclusion criteria:

(1) With other cancers or having had other cancers. (2) Underwent surgical treatment. (3) Missing clinical data. All patients were restaged according to AJCC 8th edition staging principles (9). Overall survival (OS) was defined as the time between the date of diagnosis and the date of death from any cause or the last follow-up. OS was the primary endpoint of this study.

The 462 patients who met the criteria were randomly assigned (ratio 7:3) to the training cohort and the internal validation cohort. Follow-up was performed via telephone communication with patients, with a final follow-up date of December 31, 2023. This was a retrospective study based on clinical data and did not require informed patient consent.

### Clinical parameter collection

We collected baseline clinical parameters as well as treatment regimens of patients with primary advanced non-small cell lung cancer prior to treatment. These included gender, age, weight, height, Eastern Cooperative oncology Group Performance Status (ECOG PS), smoking history, alcohol consumption history, complication (Hypertension, hyperglycemia), family history, pathology, TNM staging, chest radiotherapy, liver metastases, bone metastases, brain metastases, absolute neutrophil counts(NEUT#)(normal range: 1.80~6.30*10^9/L, platelet counts (PLT) (normal range: 125~350*10^9/L), absolute lymphocyte counts (LYMPH) (normal range:1.10~3.20*10^9/L), absolute monocyte counts (MONO) (normal range: 0.10~0.60*10^9/L), fibrinogen (FIB) (normal range: 2.00~4.00g/L), lactate dehydrogenase (LDH) (normal range:120.0~250.0U/L), D-dimer (normal range: 0~0.256mg/L), carcinoembryonic antigen (CEA) (normal range: <3.00ug/L), neuron-specific enolase (NSE) ((normal range: <12.00ug/L), squamous cell carcinoma-associated antigen (SCC) ((normal range: <1.00ng/mL), glycan antigen CA-125 ((normal range: <35.00U/mL), glycan antigen CA19-9 ((normal range: <37.00U/mL), cell proliferation index (Ki67(%)), tumor driver mutations (EGFR, MET, KRAS, ALK, ROS1, HER2, BRAF, RET, PIK3CA), treatment options. The tumor marker indicators included in this study are significant for the diagnosis of tumors and the detection of efficacy after treatment, but the effect on prognosis is not clear enough, thus we also included them in the influencing factors and tried to explore their correlation with prognosis. Elevated D-dimer may imply an increased risk of thrombosis or is associated with malignant tumors, so does it affect the patient’s prognosis, and we considered to include it in the analysis of the factors. Ki67 suggests the degree of malignancy of the tumor and is important in clinical diagnosis and prognosis, so it was included in variable selection in our study in the expectation of a more accurate determination of prognosis.

### Data analysis

**Figure 1:**
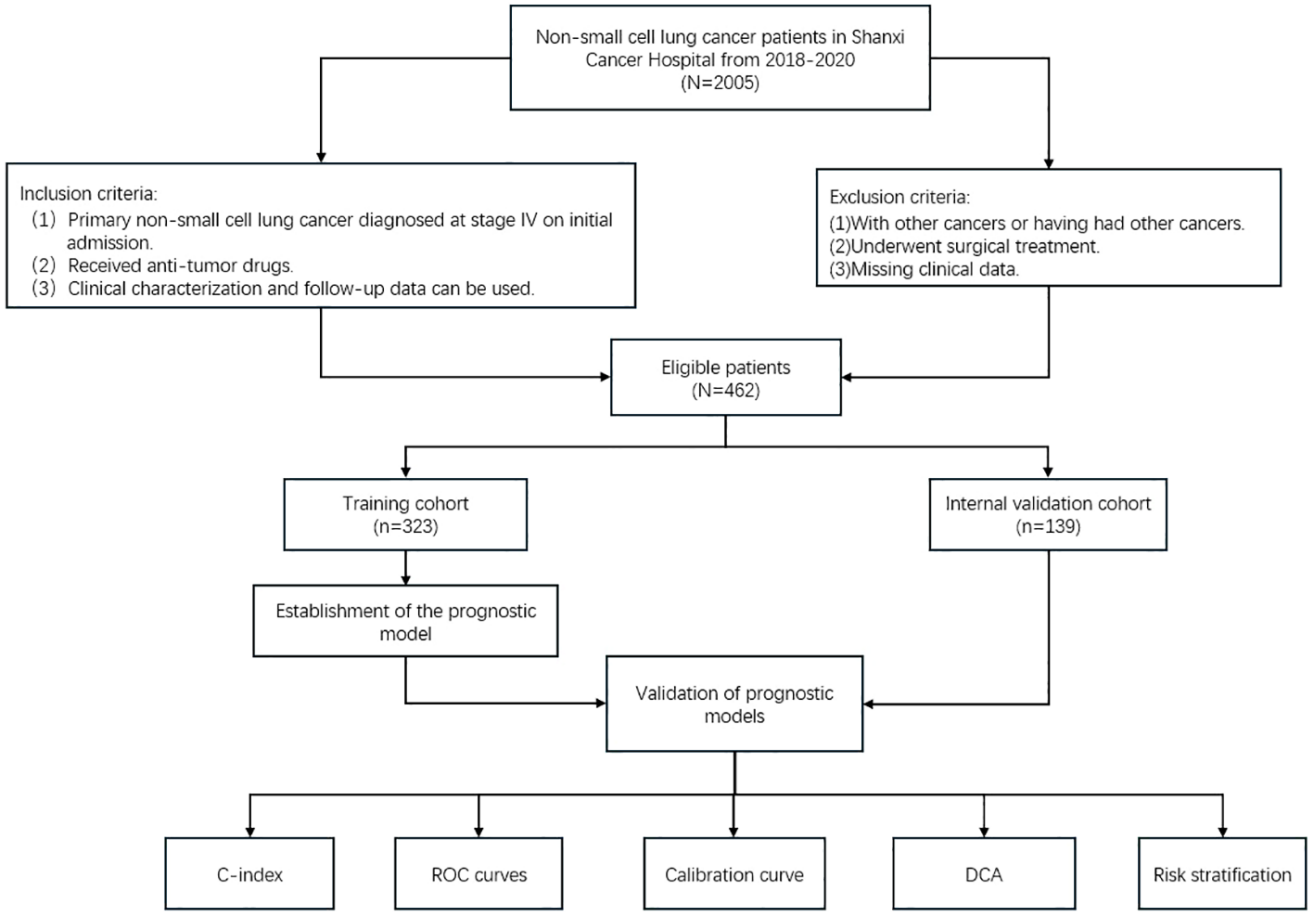
Flowchart of participant inclusion and exclusion.

All statistical analyses for this study were performed on R version 4.3.3. Patient characteristics were compared between cohorts using chi-square tests. Clinicopathological characteristics significantly associated with survival were screened using univariate and multivariate Cox regression analyses, and variables were further screened using stepwise inverse regression to select the model with the smallest Akaike Information Criterion (AIC) score as the ideal model. Finally, nomogram pre-models were constructed using the screened variables to predict the incidence of OS at 1, 2, and 3 years in patients with advanced NSCLC. We then evaluated the performance in terms of model discrimination, accuracy, and clinical application. Discriminative power was assessed using the consistency index (C-index) and the area under the subject operating characteristic curve; calibration curves measured the agreement of the probabilities generated by the nomogram plots with the actual probabilities observed. Decision curve analysis to assess the clinical utility of models. Risk scores were available for each individual in the nomogram, and risk stratification was performed for all patients using the median risk score of patients in the training cohort as a threshold. Kaplan-Meier survival analyses were performed to determine whether there were significant differences in the incidence of OS across risk groups. The flowchart for patient screening and study design is shown in **Figure 1**.

### Subgroup analysis of treatment programs

The collected patients who received chemotherapy alone and chemotherapy combined with immunotherapy were statistically paired using two-group propensity score matching, and subgroup analyses were performed among screened independent risk factors. Cox proportional risk models were used to analyze the relationship between treatment and prognosis for each subgroup. Finally, the results are displayed in a forest map.

### Analysis of the importance of variables

Importance analysis of the final variables of the model was performed using the XGBoost machine learning method. To understand the importance of the influences included in the model in the prognosis of advanced non-small cell lung cancer and to perform survival analysis on the most important variables.

## Result

### Participant characteristics

A total of 462 patients with advanced NSCLC were collected and randomly assigned to a training cohort (n = 323) and an internal validation cohort (n = 139), and there were no differences in clinicopathological and demographic characteristics between the two cohorts.

### Introduction to data characterization

Most of the 462 patients collected from Shanxi Provincial Tumor Hospital were middle-aged and elderly, with most of them concentrated between the ages of 53-85 years (75.8%), 298 (64.5%) patients were male and 164 (35.5%) were female. Epithelioid was the type of squamous carcinoma pathology in patients with a definite histologic diagnosis (17.1%). AJCC staging showed that most of the patients were T4 (32.6%) or N2 (45.2%), as it was to explore the prognosis of patients with advanced NSCLC, here we enrolled all patients with Stage IV and removed the M staging.

Laboratory data had been classified as dichotomous variables according to the reference index.

63.8% of the patients had tumor-associated mutations. 37.7% of the patients received chemotherapy alone, 29.4% of the patients received targeted therapy alone, 15.6% of the patients received chemotherapy in combination with targeted, and 17.3% of the patients received chemotherapy in combination with immunotherapy. The clinicopathologic characteristics of all patients are shown in **Table 1**.

**Table 1:** Demographics and clinicopathologic characteristics of the training and validation cohort.

Characteristics	Training cohort *(N=323)*	Validation cohort *(N=139)*	*P*.value
Sex			0.858
female	116 (35.9%)	48 (34.5%)	
male	207 (64.1%)	91 (65.5%)	
Age	61.0 [53.0;67.0]	61.0 [55.0;67.0]	0.538
Weight	63.0 [55.5;70.0]	62.0 [54.0;68.5]	0.678
Height	165 [158,170]	165 [158;170]	0.469
ECOG PS			0.220
0	2 (0.62%)	4 (2.88%)	
1	270 (83.6%)	112 (80.6%)	
2	48 (14.9%)	21 (15.1%)	
3	3 (0.93%)	2 (1.44%)	
Smoke			0.599
No	152 (47.1%)	61 (43.9%)	
Yes	171 (52.9%)	78 (56.1%)	
Drink			0.270
No	209 (64.7%)	98 (70.5%)	
Yes	114 (35.3%)	41 (29.5%)	
Complication			1.000
No	200 (61.9%)	86 (61.9%)	
Yes	123 (38.1%)	53 (38.1%)	
History			0.214
No	303 (93.8%)	135 (97.1%)	
Yes	20 (6.19%)	4 (2.88%)	
Pathology			0.844
Non-squamous carcinoma	269 (83.3%)	114 (82.0%)	
Squamous carcinoma	54 (16.7%)	25 (18.0%)	
AJCC T			0.958
1	70 (21.6%)	28 (20.1%)	
2	97 (30.0%)	45 (32.4%)	
3	51 (15.9%)	20 (14.4%)	
4	105 (32.5%)	46 (33.1%)	
AJCC N			0.712
0	49 (15.2%)	17 (12.2%)	
1	15 (4.64%)	8 (5.76%)	
2	142 (44.0%)	67 (48.2%)	
3	117 (36.2%)	47 (33.8%)	
Chest radiation			0.366
No	279 (86.4%)	125 (89.9%)	
Yes	44 (13.6%)	14 (10.1%)	
Liver metastasis			0.922
No	277 (85.8%)	118 (84.9%)	
Yes	46 (14.2%)	21 (15.1%)	
Bone metastasis			0.968
No	165 (51.1%)	72 (51.8%)	
Yes	158 (48.9%)	67 (48.2%)	
Brain metastasis			0.049
No	220 (68.1%)	108 (77.7%)	
Yes	103 (31.9%)	31 (22.3%)	
NEUT			0.305
Abnormal	81 (25.1%)	28 (20.1%)	
Normal	242 (74.9%)	111 (79.9%)	
PLT			0.905
Abnormal	73 (22.6%)	30 (21.6%)	
Normal	250 (77.4%)	109 (78.4%)	
LYMPH			0.215
Abnormal	36 (11.1%)	22 (15.8%)	
Normal	287 (88.9%)	117 (84.2%)	
MONO			0.402
Abnormal	117 (36.2%)	44 (31.7%)	
Normal	206 (63.8%)	95 (68.3%)	
FIB			0.489
Abnormal	171 (52.9%)	68 (48.9%)	
Normal	152 (47.1%)	71 (51.1%)	
LDH			0.585
Abnormal	110 (34.1%)	43 (30.9%)	
Normal	213 (65.9%)	96 (69.1%)	
D-dimer			0.847
Abnormal	193 (59.8%)	81 (58.3%)	
Normal	130 (40.2%)	58 (41.7%)	
CEA			1.000
Abnormal	203 (62.8%)	88 (63.3%)	
Normal	120 (37.2%)	51 (36.7%)	
NSE			0.552
Abnormal	17 (5.26%)	10 (7.19%)	
Normal	306 (94.7%)	129 (92.8%)	
SCC			0.521
Abnormal	41 (12.7%)	14 (10.1%)	
Normal	282 (87.3%)	125 (89.9%)	
CA199			0.642
Abnormal	80 (24.8%)	38 (27.3%)	
Normal	243 (75.2%)	101 (72.7%)	
CA125			0.167
Abnormal	150 (46.4%)	75 (54.0%)	
Normal	173 (53.6%)	64 (46.0%)	
Ki67(%)	40.0[30.0;70.0]	50.0 [30.0;60.0]	0.986
Mutation			0.369
No	112 (34.7%)	55 (39.6%)	
Yes	211 (65.3%)	84 (60.4%)	
Treatment			0.757
Alone targeted	97 (30.0%)	39 (28.1%)	
Chemotherapeutics	120 (37.2%)	54 (38.8%)	
Plus immunotherapy	53 (16.4%)	27 (19.4%)	
Plus targeted	53 (16.4%)	19 (13.7%)	

### Independent prognostic factors for screening model construction

One-way Cox regression analysis of the training cohort showed that age, ECOG PS, smoking history, alcohol consumption history, complication, pathology, N stage, liver metastasis, bone metastasis, absolute neutrophil count, platelet count, absolute lymphocyte count, absolute monocyte count, fibrinogen, lactate dehydrogenase, D-dimer, neuron-specific enolase (NSE), squamous cell carcinoma-related antigen (SCC), Ki67, tumor-associated gene mutations, and treatment regimen were significantly associated with survival (*P* < 0.05). A multifactorial analysis of the above 21 variables was performed, and the best model was determined using stepwise backward regression with the lowest AIC value. Age, ECOG PS, bone metastases, platelet count, absolute lymphocyte count,D-dimer, squamous cell carcinoma-associated antigen (SCC), Ki67, driver genes, and treatment regimen were ultimately identified as independent prognostic factors for modeling the prognosis of advanced NSCLC. The results of OS-based Cox regression survival analysis are shown in **Table 2**, respectively.

**Table 2:** Selection of variables independently associated with OS by univariate and multivariate Cox proportional hazards analysis in the training cohort.

Characteristics	Univariate			Multivariate		
	HR	CI95	*P*	HR	CI95	*P*
Age	1.02	1.01-1.04	0.001	1.03	1.01-1.04	0.001
Sex	1.29	0.98-1.70	0.075			
Weight	1.01	1.00-1.02	0.232			
Height	1.00	0.99-1.02	0.668			
ECOG PS	1.53	1.13-2.08	0.006	1.55	1.10-2.18	0.013
Smoke	1.38	1.06-1.80	0.017	0.94	0.64-1.40	0.740
Drink	1.33	1.01-1.74	0.042	1.24	0.93-1.67	0.142
Complication	1.53	1.17-2.00	0.002	1.18	0.88-1.56	0.266
History	0.53	0.27-1.04	0.064			
Pathology	1.49	1.06-2.09	0.022	0.74	0.50-1.09	0.129
AJCC T	1.05	0.94-1.17	0.418			
AJCC N	1.14	1.00-1.30	0.045	1.06	0.92-1.22	0.426
Chest radiation	0.88	0.60-1.30	0.526			
Liver metastasis	1.70	1.20-2.41	0.003	1.19	0.82-1.73	0.349
Bone metastasis	1.33	1.02-1.73	0.032	1.55	1.17-2.06	0.002
Brain metastasis	0.88	0.66-1.17	0.386			
NEUT	1.53	1.15-2.05	0.004	0.94	0.67-1.32	0.727
PLT	1.65	1.23-2.22	0.001	1.58	1.17-2.14	0.003
LYMPH	1.49	1.01-2.21	0.044	1.57	1.05-2.36	0.028
MONO	1.52	1.17-1.99	0.002	1.00	0.74-1.37	0.977
FIB	1.62	1.24-2.12	0.001	1.19	0.89-1.61	0.244
LDH	1.47	1.12-1.93	0.005	1.30	0.98-1.73	0.066
D-dimer	1.86	1.41-2.47	0.001	1.39	1.03-1.88	0.030
CEA	0.93	0.71-1.21	0.580			
NSE	2.04	1.23-3.40	0.006	1.42	0.79-2.55	0.235
SCC	1.68	1.16-2.44	0.006	1.60	1.07-2.40	0.021
CA199	1.03	0.76-1.40	0.839			
CA125	0.97	0.74-1.26	0.791			
CEA	0.93	0.71-1.21	0.580			
Ki67(%)	1.01	1.01-1.02	0.001	1.01	1.01-1.02	0.001
Mutation	0.56	0.43-0.73	0.001	0.51	0.38-0.69	0.001
Treatment	0.75	0.66-0.86	0.001	0.72	0.63-0.83	0.001

R, hazard ratio; CI95, 95% confidence interval; AJCC Stages, the eighth edition American Joint Committee on Cancer (AJCC) TNM staging system.

### Model creation and validation

**Figure 2:**
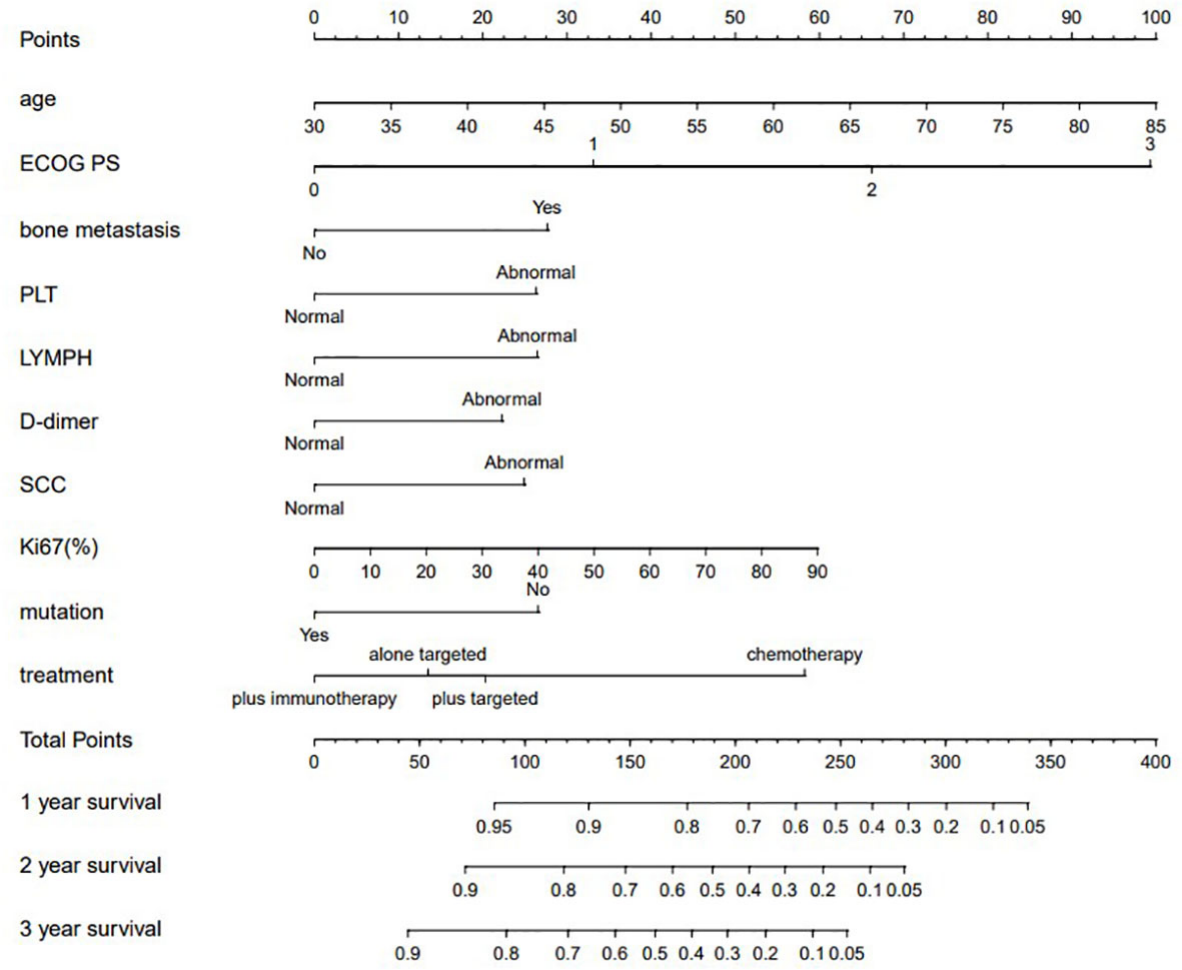
Nomograms for predicting 1, 2, and 3years OS of patients with advanced NSCLC.

The model for predicting late survival in NSCLC patients was determined by the ten variables screened above and visualized in a nomogram (**Figure 2**). By calculating the sum of the scores of the ten variables from the nomogram, we can estimate the OS rates of advanced NSCLC patients at 1, 2, and 3 years. The performance of the model was validated using the C-index, ROC curve over time and calibration curve. The C-index of the OS-based prediction model was 0.711 (95% CI, 0.677-0.743) and 0.696 (95% CI, 0.614-0.717) for the training group and the internal validation group, respectively.

**Figure 3:**
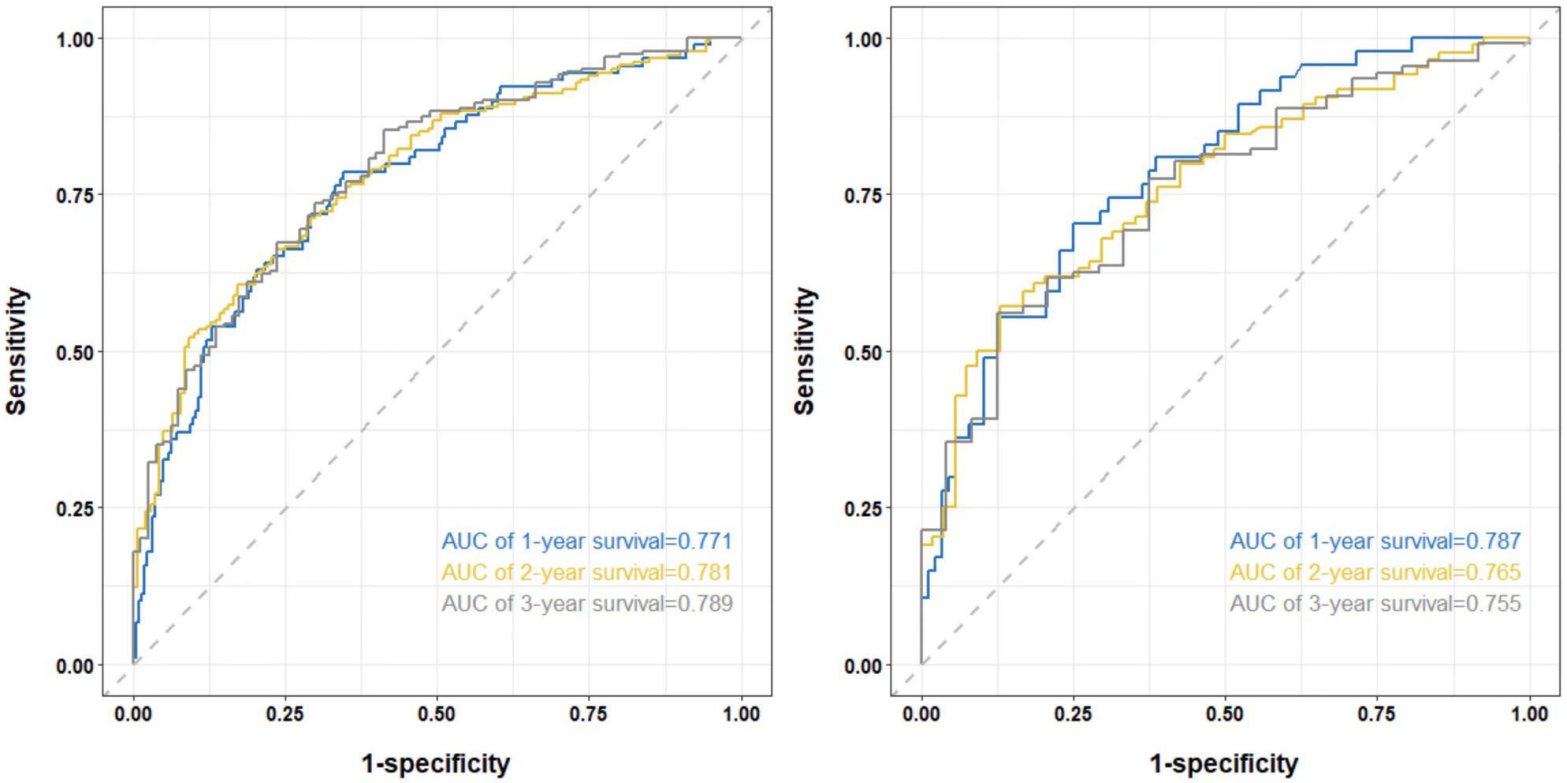
The time-dependent ROC curves of the nomogram predicting OS at (left) 1-year and 2-year and 3-year in the training cohort, and at (right) 1-year and 2-year and 3-year in the internal validation cohort.

**Figure 3** shows the AUC values of the column-line plots of predicted 1-, 2-, and 3-year OS in the two cohorts Training cohort: 1-year OS 0.771 (95% CI, 0.713-0.830); 2-year OS 0.781 (95% CI, 0.732-0.831); 3-year OS 0.789 (95% CI, 0.733-0.844); internal validation cohort: 1-year OS.

0.787 (95% CI, 0.709-0.865); 2-year OS 0.765 (95% CI, 0.686 -0.843); 3-year OS 0.755 (95% CI, 0.656-0.854). The C-index and the AUC values indicated that the prognostic model had a better discriminative ability for survival in advanced NSCLC patients.

**Figure 4:**
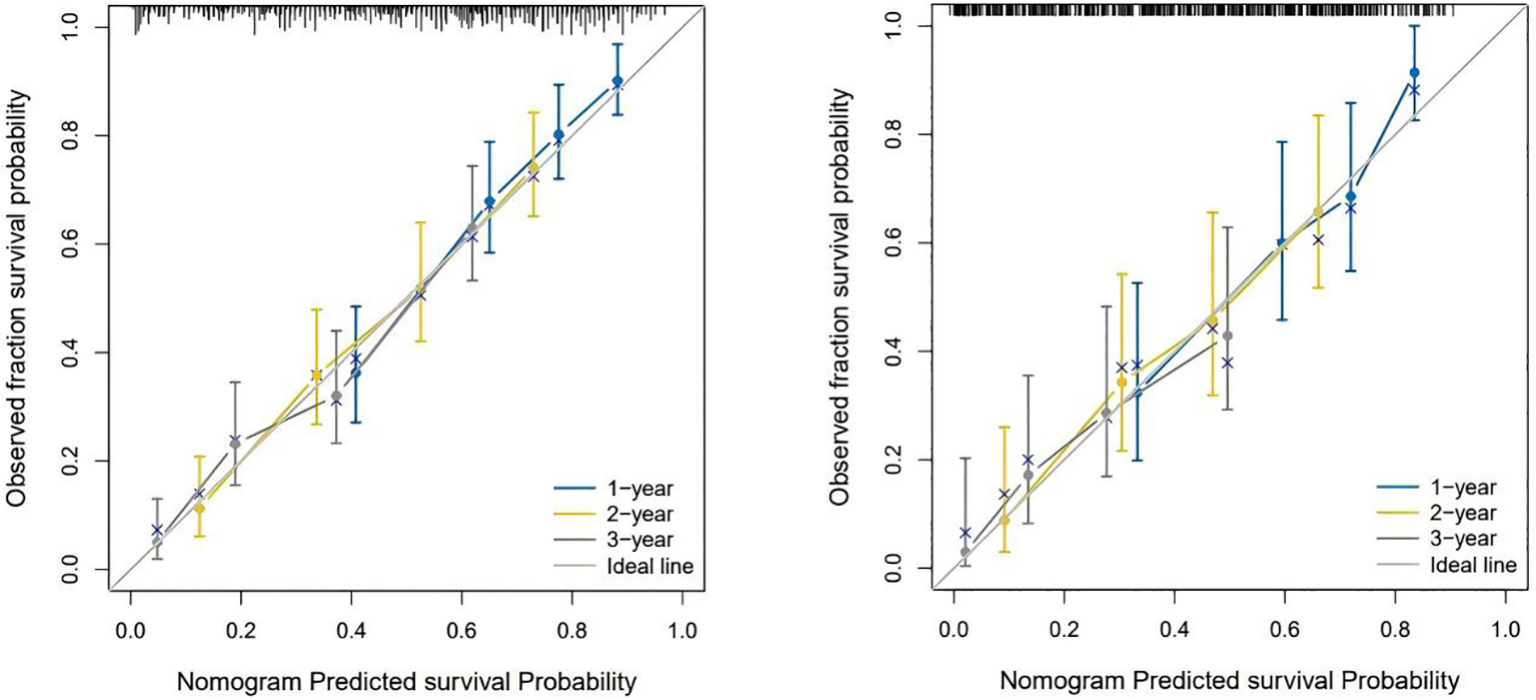
The calibration curves for predicting OS at (left) 1-year and 2-year and 3-year in the training cohort, and at (right) 1-year 2-year and 3- year in the internal validation cohort.

**Figure 4** shows the calibration curves of the prognostic model between the actual OS rates and the predicted probabilities of the two cohorts at 1, 2, and 3 years, respectively, demonstrating that the survival rates generated by the nomogram are in good agreement with those observed in the actual population.

**Figure 5:**
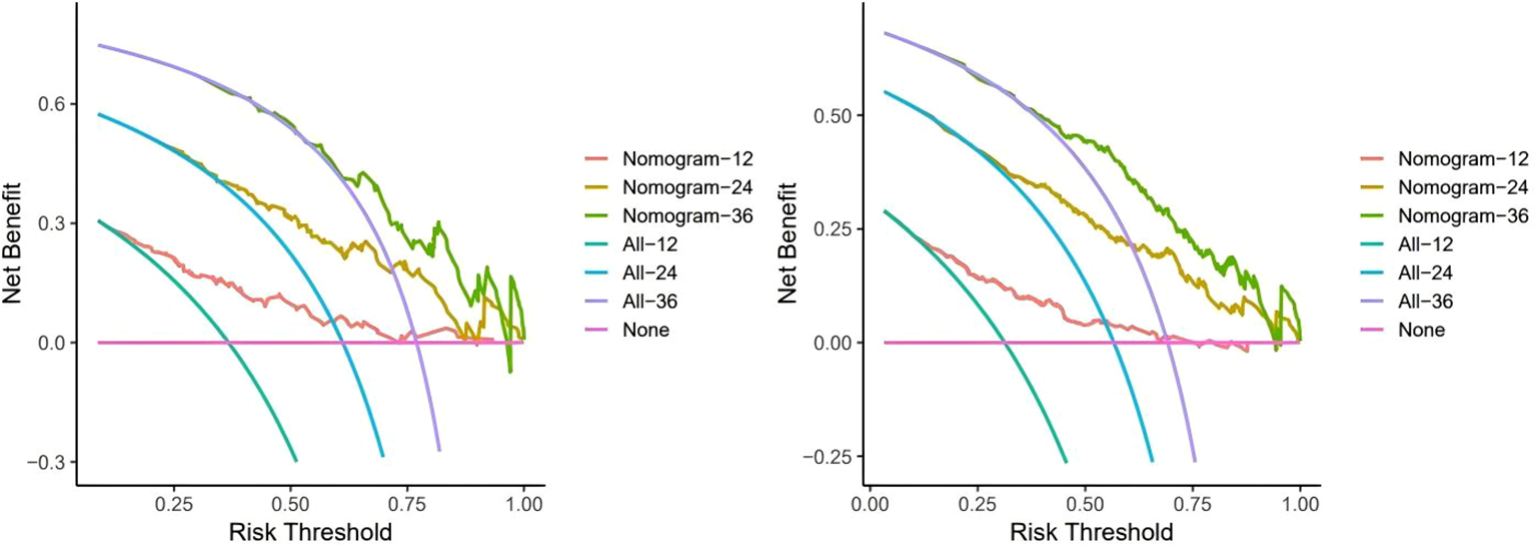
Decision curve analysis to assess clinical benefit. Here we use a monthly unit to record the benefits over three years. The training cohort (left) Internal validation cohort (right).

**Figure 5** shows the clinical benefits of the constructed model at 1, 2, and 3 years in both cohorts, suggesting that the model can achieve good benefits in clinical applications.

### Risk stratification based on nomogram

**Figure 6:**
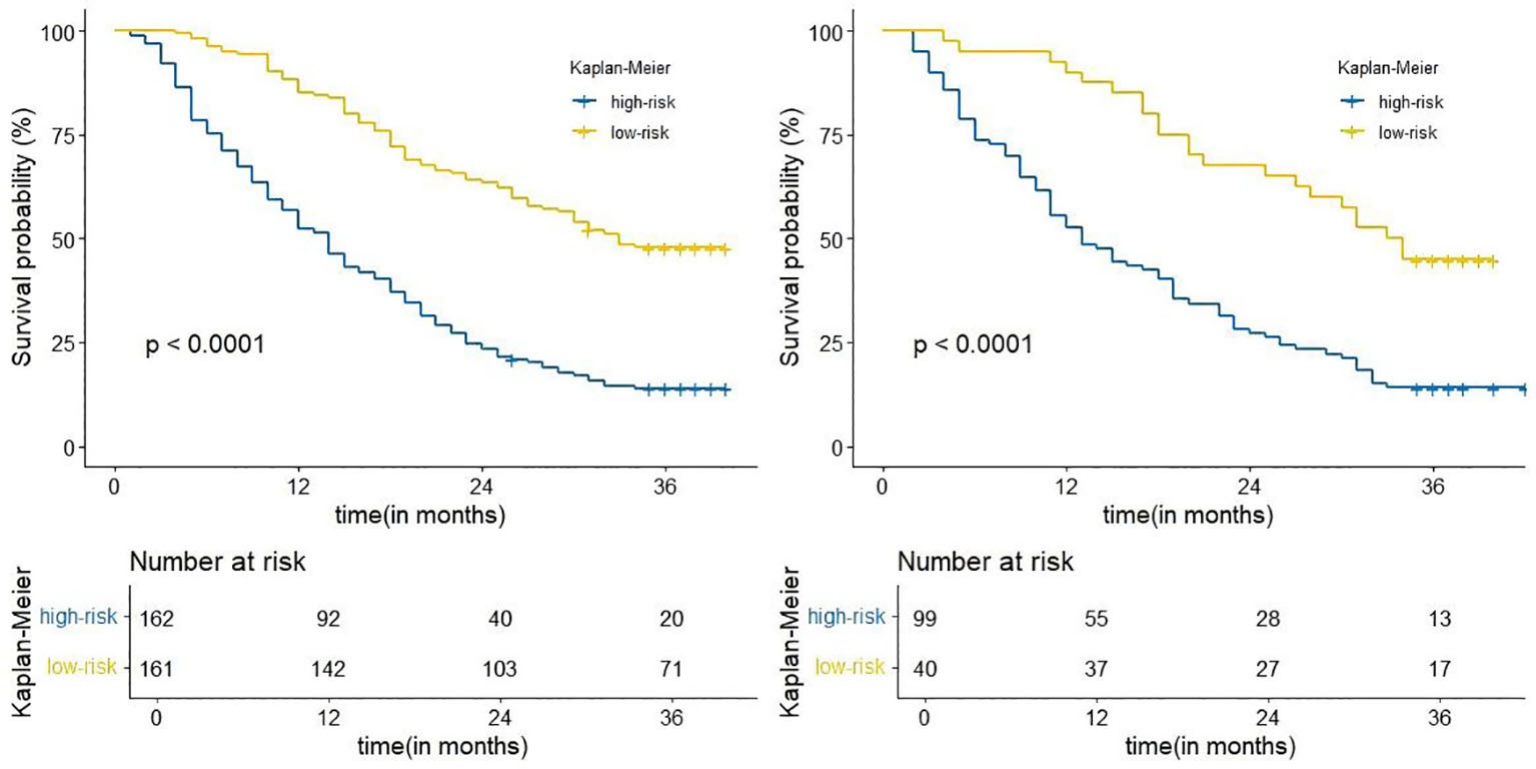
Kaplan-Meier curves for correlation with OS for the low and high-risk groups in the training cohort (left), internal validation cohort (right).

Risk scores were calculated for all patients by nomogram, and the median risk score of the training cohort (OS: 205.3) was used as the threshold for categorizing patients into high-risk (OS:

risk score ≥205.3) and low-risk groups (OS: risk score <205.3).The Kaplan-Meier survival analysis showed a significant difference in OS between different risk groups (**Figure 6**), suggesting that column line plotting can help us accurately stratify the risk of patients with advanced NSCLC.

### Prognostic value of immunotherapy in advanced NSCLC

**Figure 7:**
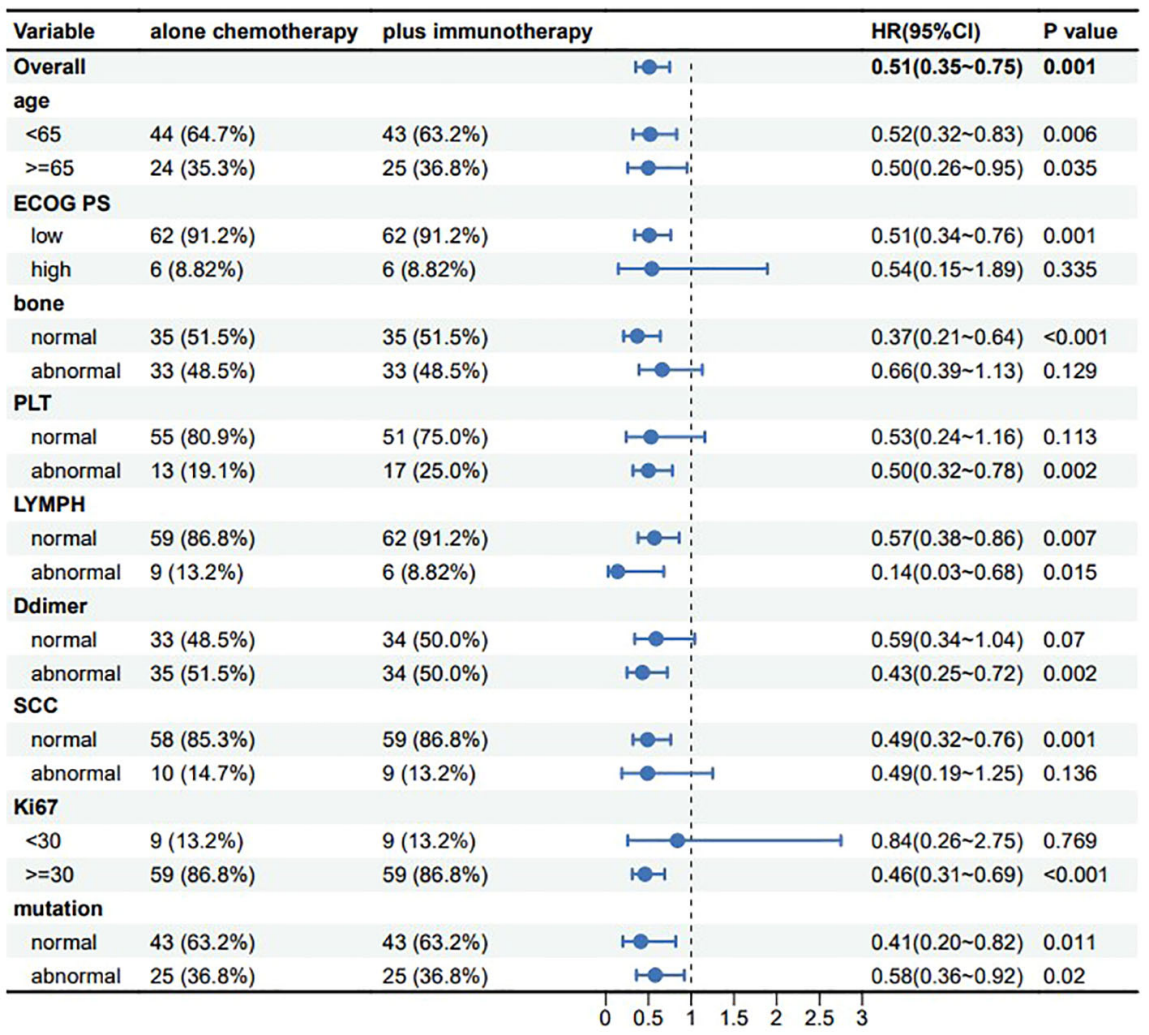
Subgroup analysis of chemotherapy and chemotherapycombined immunotherapy.

With the development of innovative drugs, the use of immunotherapy in NSCLC patients is gradually increasing. To investigate the prognostic value of immunotherapeutic agents in patients with advanced NSCLC, we performed a controlled analysis of chemotherapy regimens combined with immunotherapy versus chemotherapy regimens alone. In our study, the R language MatchIt package was used for propensity score matching analysis. A 1:1 greedy nearest neighbor matching with a PS score of 0.1 was used to derive pairs of patients receiving chemotherapy combined with immunotherapy and chemotherapy only. Matching variables which include age, ECOG PS, bone metastasis, PLT, LYMPH, SCC, D-dimer, Ki67, and tumor driver gene mutations are nine variables. This strategy resulted in 68 matched pairs in each group, for a total of 136 patients included in the subgroup analysis. It was evident from the results that patients receiving chemotherapy combined with immunotherapy tended to have better OS in all subgroups, and all results were statistically significant (**Figure 7**), suggesting that the addition of immunotherapeutic agents to chemotherapy can provide a survival benefit for patients with advanced NSCLC.

### Importance analysis of model variables

**Figure 8:**
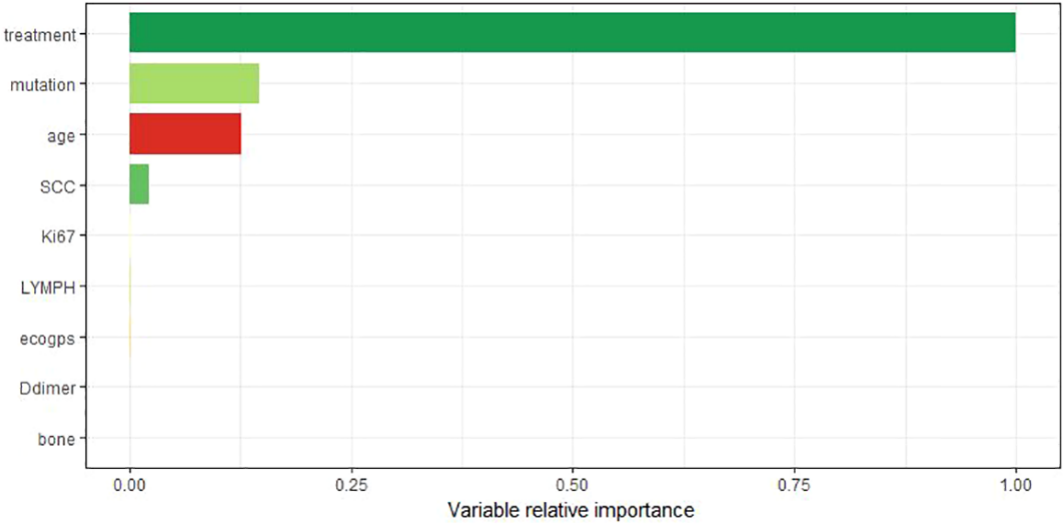
The importance ranking of variables takes the most important variable as the reference value.

**Figure 9:**
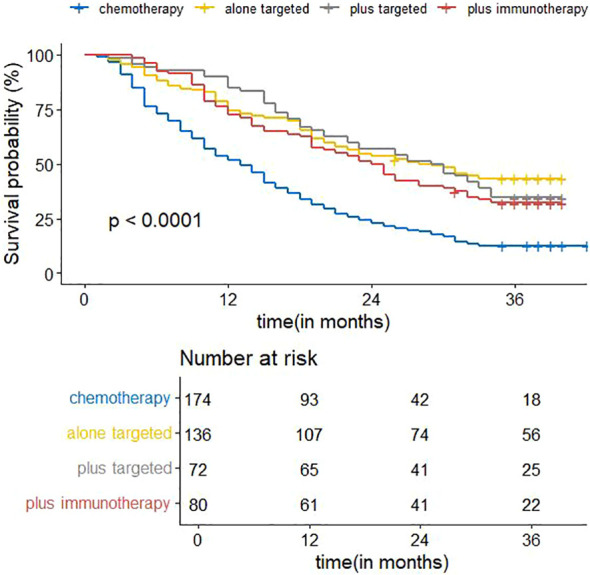
Kaplan-Meier curves for correlation with OS for the four treatment regimen.

**Figure 8** shows the visualization results of the ordering of the importance of the model variables, in which the treatment regimen accounts for the highest percentage, indicating that the treatment regimen is the most important among all the variables of the model, and that the treatment regimen is the most critical factor among the influencing factors of the survival prognosis of patients with advanced non-small cell lung cancer. Therefore, we analyzed the survival curves for all patients’ treatment regimens (**Figure 9**). The results showed that targeted therapy alone, chemotherapy combined with targeted therapy and chemotherapy combined with immunotherapy all had better survival than chemotherapy alone.

## Discussion

Advanced non-small cell lung cancer is one of the most common types of lung cancer, and thus its data are more readily available. We collected a large number of patient samples from Shanxi Cancer Hospital, which facilitated our study of advanced non-small cell lung cancer.

In this work, we constructed a prognostic model based on basic information as well as clinical characteristics of patients with advanced non-small cell lung cancer. 462 patients from Shanxi Provincial Cancer Hospital were randomly assigned to the training cohort and the internal validation cohort, and were screened by Cox’s one-way analysis for, age, ECOG PS, history of smoking, history of alcohol, complication, pathology, N-stage, liver metastasis, bone metastasis, absolute neutrophil value, platelet count, absolute lymphocyte value, absolute monocyte value, fibrinogen, lactate dehydrogenase, D-dimer, neuron-specific enolase (NSE), squamous cell carcinoma-associated antigen (SCC), Ki67, tumor-associated gene mutations, and treatment regimen were significantly associated with survival.

With the increase of age, the risk of death of patients increases (10). For middle-aged and elderly people, the function of human organs gradually decreases with age, and the invasion of non-small cell lung cancer accelerates this process, which seriously affects the survival and prognosis of patients.

Previous studies have shown that ECOG PS is an independent factor affecting prognosis (11, 12), which is consistent with the results of the present study, in which patients were subjected to tumor invasion resulting in a decreased physical activity status, compromised survival, and consequently a poor prognosis.

Bone metastasis is one of the prevalent metastases in advanced NSCLC patients, and it is also the most important factor leading to poor quality of life and low survival rate of lung cancer patients (13). Patients with bone metastases are often accompanied by severe bone pain, which seriously affects the quality of survival of patients.

In laboratory tests of patients with advanced NSCLC, platelets and lymphocytes were found to be independent influences on the survival prognosis of patients, and abnormalities in these two indices suggested a poor prognosis. Clemens Hinterleitner et al. found that platelets interact with lung cancer cells and transfer PD-L1 from tumor cells to platelets (14) suggesting that platelets are associated with tumor immune escape mechanisms thereby affecting patient prognosis. A study by Li Xiaohui et al. showed that platelets can promote the growth of lung adenocarcinoma (15). This further establishes that platelets are an independent influence on the prognosis of non-small cell lung cancer. In the study of Yoon Ya-Nam et al. neutrophil to lymphocyte ratio affects survival in patients with advanced non-small cell lung cancer (16). In this study although neutrophils to lymphocytes in the form of a ratio was not used as a variable, lymphocytes were still identified as an independent prognostic factor.

Previous studies have shown that D-dimer is associated with poor prognosis in non-small cell lung cancer (17–19), which is consistent with the findings of this study. Cancer cells usually regulate coagulation and fibrinolysis in cancer patients, and elevated plasma D-dimer suggests that patients may have a hypercoagulable state of the blood or thrombosis, which can cause damage to the organism leading to a poor prognosis.

Serum tumor markers (STMs) are circulating protein molecules produced by tumor cells or other cells in the body in response to cancer or certain benign diseases. Changes in their serum levels have been shown to reflect tumor quality, making them valuable in predicting prognosis and assessing response to treatment during follow-up. Results have shown that CA 125 antigen (CA-125), carcinoembryonic antigen (CEA), cytokeratin 19 fragment (Cyfra 21.1) and squamous cell carcinoma antigen (SCC-Ag) are associated with NSCLC disease (20, 21). In this study squamous cell carcinoma antigen (SCC-Ag) was found to be a prognostic influencing factor in non-small cell lung cancer, when its value was elevated suggesting a poor prognosis for the patient.

Ki67 has significant clinical value in the treatment and prognosis of NSCLC (22, 23). Ki67 is an associated antigen of value-added cells, and its function is closely related to mitosis, which is indispensable in cell proliferation. The higher the proliferation index of Ki67, the higher the cell proliferation ability, the higher the degree of malignancy, and the worse the patient’s prognosis.

Several studies have shown that mutations in tumor-associated genes affect the prognosis of patients with non-small cell lung cancer (24–28). In current therapeutic decisions, whether a tumor-associated gene is mutated or not is a key factor that can guide clinically available targeted therapies. We are rapidly discovering that more and more mutations occur in targetable pathways, and targeted therapies have dramatically altered treatment outcomes and disease prognosis (29).

Treatment regimen is significantly associated with survival prognosis in patients with advanced NSCLC (30). The importance of treatment regimen in the prognosis of advanced NSCLC can be seen in our variable significance analyses. The 2024 version of the Clinical Practice Guidelines in Oncology suggests that different treatment regimens should be chosen for patients with advanced or metastatic NSCLC, depending on their oncogenic drivers. For example, NSCLC with EGFR alterations is usually treated with targeted agents (gefitinib, ositinib, etc.) as the first line of therapy (31).

The emergence of immuno-oncology has revolutionized the treatment of metastatic non-small cell lung cancer (32). In recent years, immunotherapy has been increasingly used in the treatment of non-small cell lung cancer, and several clinical studies have shown that receiving immunotherapy can increase survival and effectively improve the prognosis of patients (33–35). Does immunotherapy still perform satisfactorily in the real world? Our subgroup analysis of patients receiving chemotherapy alone and chemotherapy combined with immunotherapy in the treatment regimen group showed that there was a significant difference in prognosis between patients receiving systemic chemotherapy and chemotherapy combined with immunotherapy in the advanced stages, with the group of patients with the addition of immunotherapy having a higher survival than the group receiving chemotherapy alone. Survival analysis plots of the treatment regimens also showed that immunotherapy improved survival, with a median survival time of 13 months for patients receiving chemotherapy alone and 24.5 months for patients receiving chemotherapy combined with immunotherapy. Immunotherapy combined with chemotherapy has a better therapeutic effect. Systemic chemotherapy, because of its lack of specificity, will damage normal body cells at the same time as it has a killing effect on the tumor, thus it is inefficient and produces serious adverse reactions, which may be the reason for the poor prognosis of the patients. When chemotherapy is combined with immunotherapy, the immune drug effectively improves the body’s immune function, eliminates the escape mechanism of tumor cells, so that the tumor cells can be recognized by the body, which greatly improves the killing effect on the tumor cells, and effectively prolongs the survival of patients. It is recommended that immunotherapy be incorporated more into clinical regimens, which may benefit more patients with advanced non-small cell lung cancer. In addition, radiation therapy, as an adjuvant treatment, also occupies a certain position in patients with advanced non-small cell lung cancer, but the correlation with prognosis was not reflected in this study, and it is suggested that radiation therapy can be used as a palliative treatment to relieve localized pain, but symptomatic improvement may not be converted into OS benefit.

The data used in this study were collected from real-world clinical data, which can truly reflect the status of patients with advanced non-small cell lung cancer, and therefore have greater reference value. We hope that this model can provide a reference for clinical treatment, and that the construction of such models will help to discover new tumor-related prognostic factors. Due to regional limitations, we did not collect enough external data to serve as an external validation group, so this study lacks external validation of the model, and the extrapolation ability of the model is unknown. We hope that more internal and external cases can be collected subsequently to validate and optimize the model and better correct the model performance.

## Conclusion

In summary, we successfully constructed and validated a prognostic model to predict the survival rate of patients with advanced NSCLC, which provides a more accurate basis for the treatment decision of such patients. Systemic chemotherapy dominates in advanced NSCLC patients, and chemotherapy combined with immunotherapy can improve the survival probability of advanced NSCLC patients, and it is suggested that immunotherapy should be incorporated into clinical treatment protocols more frequently.

## Data Availability

The original contributions presented in the study are included in the article/**Supplementary Material**. Further inquiries can be directed to the corresponding author.

## References

[1] Hendriks LEL Remon J Faivre-Finn C Garassino MC Heymach JV Kerr KM. Non-small-cell lung cancer. Nat Rev Dis Primers. (2024) 10:71. doi: 10.1038/s41572-024-00551-9 39327441

[2] Manjunath Y Mitchem JB Suvilesh KN Avella DM Kimchi ET Staveley-O'Carroll KF. Circulating giant tumor-macrophage fusion cells are independent prognosticators in patients with NSCLC. J Thorac Oncol. (2020) 15:1460–71. doi: 10.1016/j.jtho.2020.04.034 32416323

[3] Siegel RL Miller KD Wagle NS Jemal A. Cancer statistics, 2023. CA Cancer J Clin. (2023) 73:17–48. doi: 10.3322/caac.21763 36633525

[4] Herbst RS Morgensztern D Boshoff C. The biology and management of non-small cell lung cancer. Nature. (2018) 553:446–54. doi: 10.1038/nature25183 29364287

[5] Mouritzen MT Junker KF Carus A Ladekarl M Meldgaard P Nielsen AWM. Clinical features affecting efficacy of immune checkpoint inhibitors in pretreated patients with advanced NSCLC: a Danish nationwide real-world study. Acta Oncol. (2022) 61:409–16. doi: 10.1080/0284186X.2021.2023213 35012430

[6] Li Y Juergens RA Finley C Swaminath A. Current and future treatment options in the management of stage III NSCLC. J Thorac Oncol. (2023) 18:1478–91. doi: 10.1016/j.jtho.2023.08.011 37574133

[7] Duma N Santana-Davila R Molina JR. Non-small cell lung cancer: epidemiology, screening, diagnosis, and treatment. Mayo Clin Proc. (2019) 94:1623–40. doi: 10.1016/j.mayocp.2019.01.013 31378236

[8] Wang M Herbst RS Boshoff C. Toward personalized treatment approaches for non-small-cell lung cancer. Nat Med. (2021) 27:1345–56. doi: 10.1038/s41591-021-01450-2 34385702

[9] Heymach JV Harpole D Mitsudomi T Taube JM Galffy G Hochmair M. Perioperative durvalumab for resectable non-small-cell lung cancer. N Engl J Med. (2023) 389:1672–84. doi: 10.1056/NEJMoa2304875 37870974

[10] Huang X Wu S Chen S Qiu M Zhao Y Wei J. Prognostic impact of age in advanced non-small cell lung cancer patients undergoing first-line checkpoint inhibitor immunotherapy and chemotherapy treatment. Int Immunopharmacol. (2024) 132:111901. doi: 10.1016/j.intimp.2024.111901 38554448

[11] Cunha MT De Souza Borges AP Carvalho Jardim V Fujita A de Castro G Jr. Predicting survival in metastatic non-small cell lung cancer patients with poor ECOG-PS: A single-arm prospective study. Cancer Med. (2023) 12:5099–109. doi: 10.1002/cam4.v12.4 PMC9972023 36161783

[12] Prelaj A Galli EG Miskovic V Pesenti M Viscardi G Pedica B. Real-world data to build explainable trustworthy artificial intelligence models for prediction of immunotherapy efficacy in NSCLC patients. Front Oncol. (2022) 12:1078822. doi: 10.3389/fonc.2022.1078822 36755856 PMC9899835

[13] Zhao MN Zhang LF Sun Z Qiao L-H Yang T Ren Y-Z. A novel microRNA-182/Interleukin-8 regulatory axis controls osteolytic bone metastasis of lung cancer. Cell Death Dis. (2023) 14:298. doi: 10.1038/s41419-023-05819-8 37127752 PMC10151336

[14] Hinterleitner C Strähle J Malenke E Hinterleitner M Henning M Seehawer M. Platelet PD-L1 reflects collective intratumoral PD-L1 expression and predicts immunotherapy response in non-small cell lung cancer. Nat Commun. (2021) 12:7005. doi: 10.1038/s41467-021-27303-7 34853305 PMC8636618

[15] Li X Li M Hu Z Zhou L Zheng M Jiao D. Tumor-infiltrating platelets promote the growth of lung adenocarcinoma. Transl Oncol. (2024) 39:101813. doi: 10.1016/j.tranon.2023.101813 38235621 PMC10628888

[16] Wan Y-N Chen H-M Liu X-F Gu W-G Lu Y-Y. Elevated pretreatment neutrophil-to-lymphocyte ratio indicate low survival rate in apatinib-treated patients with non-small cell lung cancer: A STROBE-compliant article. Med (Baltimore). (2022) 101:e32043. doi: 10.1097/MD.0000000000032043 PMC9704969 36451494

[17] Chang F Zhang H Chen C Ke Z Zhao M Fan X. Concomitant genetic alterations are associated with plasma D-dimer level in patients with non-small-cell lung cancer. Future Oncol. (2022) 18:679–90. doi: 10.2217/fon-2021-0455 34789015

[18] Chen C Yin H Zhang Y Chen H Xu J Ren L. Plasma D-dimer and interleukin-6 are associated with treatment response and progression-free survival in advanced NSCLC patients on anti-PD-1 therapy. Cancer Med. (2023) 12:15831–40. doi: 10.1002/cam4.v12.15 PMC10469714 37326149

[19] Guo J Gao Y Gong Z Dong P Mao Y Li F. Plasma D-dimer level correlates with age, metastasis, recurrence, tumor-node-metastasis classification (TNM), and treatment of non-small-cell lung cancer (NSCLC) patients. BioMed Res Int. (2021) 2021:9623571. doi: 10.1155/2021/9623571 34712737 PMC8548094

[20] Vos D Rao S Pierce JD Smith DA Tirumani SH Yoest JM. The past, present, and future (Liquid biopsy) of serum tumor markers in lung cancer: A primer for the radiologist. J Comput Assist Tomogr. (2021) 45:950–8. doi: 10.1097/RCT.0000000000001204 34347703

[21] Wang L Wang D Zheng G Yang Y Du L Dong Z. Clinical evaluation and therapeutic monitoring value of serum tumor markers in lung cancer. Int J Biol Markers. (2016) 31:e80–7. doi: 10.5301/jbm.5000177 26560853

[22] Hu D Li X Lin C Wu Y Jiang H. Deep learning to predict the cell proliferation and prognosis of non-small cell lung cancer based on FDG-PET/CT images. Diagnost (Basel). (2023) 1331. doi: 10.3390/diagnostics13193107 PMC10573026 37835850

[23] Palumbo B Capozzi R Bianconi F Fravolini ML Cascianelli S Messina SG. Classification model to estimate MIB-1 (Ki 67) proliferation index in NSCLC patients evaluated with (18)F-FDG-PET/CT. Anticancer Res. (2020) 40:3355–60. doi: 10.21873/anticanres.14318 32487631

[24] Ciardiello F Hirsch FR Pirker R Felip E Valencia C Smit EF. The role of anti-EGFR therapies in EGFR-TKI-resistant advanced non-small cell lung cancer. Cancer Treat Rev. (2024) 122:102664. doi: 10.1016/j.ctrv.2023.102664 38064878

[25] Hu M Zhong C Wang J Chen JQ Zhou T. Current status and breakthroughs in treating advanced non-small cell lung cancer with EGFR exon 20 insertion mutations. Front Immunol. (2024) 15:1399975. doi: 10.3389/fimmu.2024.1399975 38774882 PMC11106363

[26] Pan D Hu AY Antonia SJ Li C-Y. A gene mutation signature predicting immunotherapy benefits in patients with NSCLC. J Thorac Oncol. (2021) 16:419–27. doi: 10.1016/j.jtho.2020.11.021 PMC7920921 33307194

[27] Yang SR Schultheis AM Yu H Mandelker D Ladanyi M Buttner R. Precision medicine in non-small cell lung cancer: Current applications and future directions. Semin Cancer Biol. (2022) 84:184–98. doi: 10.1016/j.semcancer.2020.07.009 32730814

[28] Zhang W Lin X Li X Wang M Sun W Han X. Survival prediction model for non-small cell lung cancer based on somatic mutations. J Gene Med. (2020) 22:e3206. doi: 10.1002/jgm.v22.9 32367667

[29] Waarts MR Stonestrom AJ Park YC Levine RL. Targeting mutations in cancer. J Clin Invest. (2022) 132(8):e154943. doi: 10.1172/JCI154943 35426374 PMC9012285

[30] Banna GL Cantale O Muthuramalingam S Cave J Comins C Cortellini A. Efficacy outcomes and prognostic factors from real-world patients with advanced non-small-cell lung cancer treated with first-line chemoimmunotherapy: The Spinnaker retrospective study. Int Immunopharmacol. (2022) 110:108985. doi: 10.1016/j.intimp.2022.108985 35777264

[31] Riely GJ Wood DE Ettinger DS Aisner DL Akerley W Bauman JR. Non-small cell lung cancer, version 4.2024, NCCN clinical practice guidelines in oncology. J Natl Compr Canc Netw. (2024) 22:249–74. doi: 10.6004/jnccn.2204.0023 38754467

[32] Desai A Peters S. Immunotherapy-based combinations in metastatic NSCLC. Cancer Treat Rev. (2023) 116:102545. doi: 10.1016/j.ctrv.2023.102545 37030062

[33] Reck M Remon J Hellmann MD. First-line immunotherapy for non-small-cell lung cancer. J Clin Oncol. (2022) 40:586–97. doi: 10.1200/JCO.21.01497 34985920

[34] Reck M Rodríguez-Abreu D Robinson AG Hui R Csoszi T Fulop A. Five-year outcomes with pembrolizumab versus chemotherapy for metastatic non-small-cell lung cancer with PD-L1 tumor proportion score ≥ 50. J Clin Oncol. (2021) 39:2339–49. doi: 10.1200/JCO.21.00174 PMC8280089 33872070

[35] Waterhouse D Lam J Betts KA Yin L Gao S Yuan Y. Real-world outcomes of immunotherapy-based regimens in first-line advanced non-small cell lung cancer. Lung Cancer. (2021) 156:41–9. doi: 10.1016/j.lungcan.2021.04.007 33894493

